# Successful salvage surgery following multimodal therapy in a patient who harboured ALK‐rearranged advanced lung adenocarcinoma with multiple organ metastases

**DOI:** 10.1002/rcr2.451

**Published:** 2019-06-24

**Authors:** Yoshitsugu Horio, Tetsuya Mizuno, Yukinori Sakao, Yoshitaka Inaba, Yasushi Yatabe, Toyoaki Hida

**Affiliations:** ^1^ Department of Outpatient Services Aichi Cancer Center Hospital Nagoya Japan; ^2^ Department of Thoracic Oncology Aichi Cancer Center Hospital Nagoya Japan; ^3^ Department of Thoracic Surgery Aichi Cancer Center Hospital Nagoya Japan; ^4^ Department of Diagnostic and Interventional Radiology Aichi Cancer Center Hospital Nagoya Japan; ^5^ Department of Pathology and Molecular Diagnostics Aichi Cancer Center Hospital Nagoya Japan

**Keywords:** ALK‐TKI, lung cancer, pemetrexed, salvage surgery, treatment‐free remission

## Abstract

The prognosis of stage IVb non‐small cell lung cancer (NSCLC) patients with multiple distant metastases or involvement of different extra‐thoracic sites is poor. The prognosis following salvage surgery for patients with more than five metastases has been reported as most unfavourable. The following case is of a 71‐year‐old man with a 9‐year survival duration after being diagnosed with stage IVb ALK‐rearranged lung adenocarcinoma, who was treated for 6 years with whole‐brain radiotherapy, pemetrexed‐based chemotherapy, ALK‐tyrosine kinase inhibitors (TKIs) including ceritinib and alectinib, and salvage sublobar resection of the primary lung cancer and who obtained treatment‐free remission (TFR) for more than 3 years following surgery.

## Introduction

Salvage surgery in non‐small cell lung cancer (NSCLC) is defined as the surgical resection of persistent or recurrent primary lung tumours after previous local treatments, including chemo‐ and/or radiotherapy for locally advanced lung cancer and ablative radiotherapy for stage I lung cancer, in addition to urgent resection for patients with haemoptysis or severe infections 1. However, complete tumour resection in selected cases of stages IIIA–IV advanced NSCLC can be achieved and contribute to the prolongation of life. Indeed, stage IVa NSCLC patients with a single metastasis in a single extra‐thoracic organ, such as the brain or adrenal gland, are good candidates for aggressive local ablative therapy to both the primary thoracic and metastatic site of disease 2. Although a surgical cure is rare, the indications for salvage surgery in advanced NSCLC are extending as emerging therapies, including pemetrexed‐based chemotherapy, bevacizumab, ramucirumab, tyrosine kinase inhibitors (TKIs), and immune checkpoint inhibitors in NSCLC, have demonstrated significant efficacy in survival 2.

In this paper, an ALK‐positive advanced lung cancer patient with multiple organ metastases was successfully treated with salvage sublobar resection after multimodal treatments and obtained treatment‐free remission (TFR) for more than 3 years.

## Case Report

**Figure 1:**
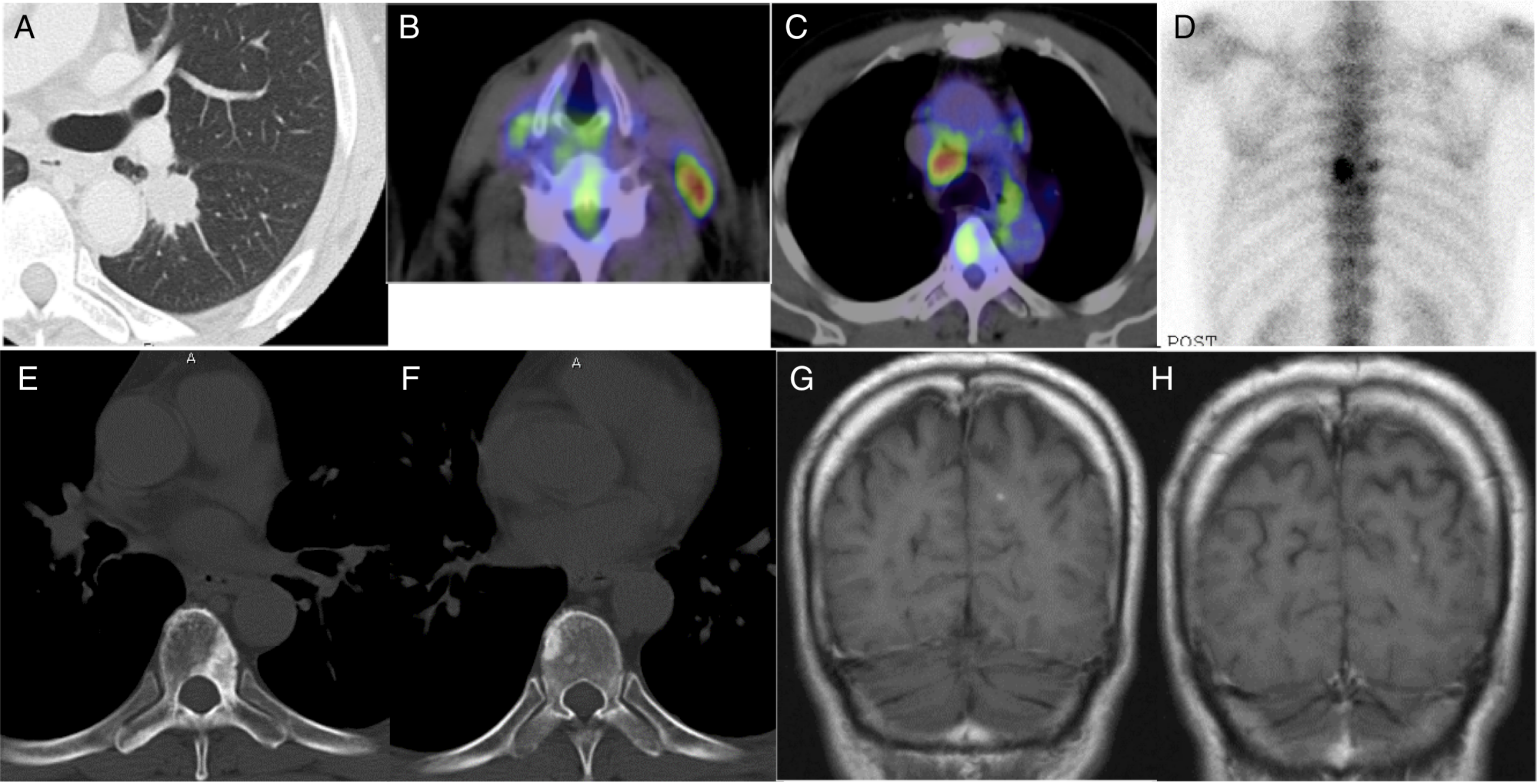
Images prior to commencing treatments: (A) Computed tomography (CT) demonstrated lung tumour of the left lower lobe. (B, C) positron emission tomography (PET)‐CT showed Fluorodeoxyglucose (FDG) uptake of left cervical and contralateral mediastinal lymph nodes. (D, E, F) Bone scintigraphy and CT demonstrated vertebral metastases. (G) Magnetic resonance imaging showed multiple small brain metastases.

**Figure 2:**
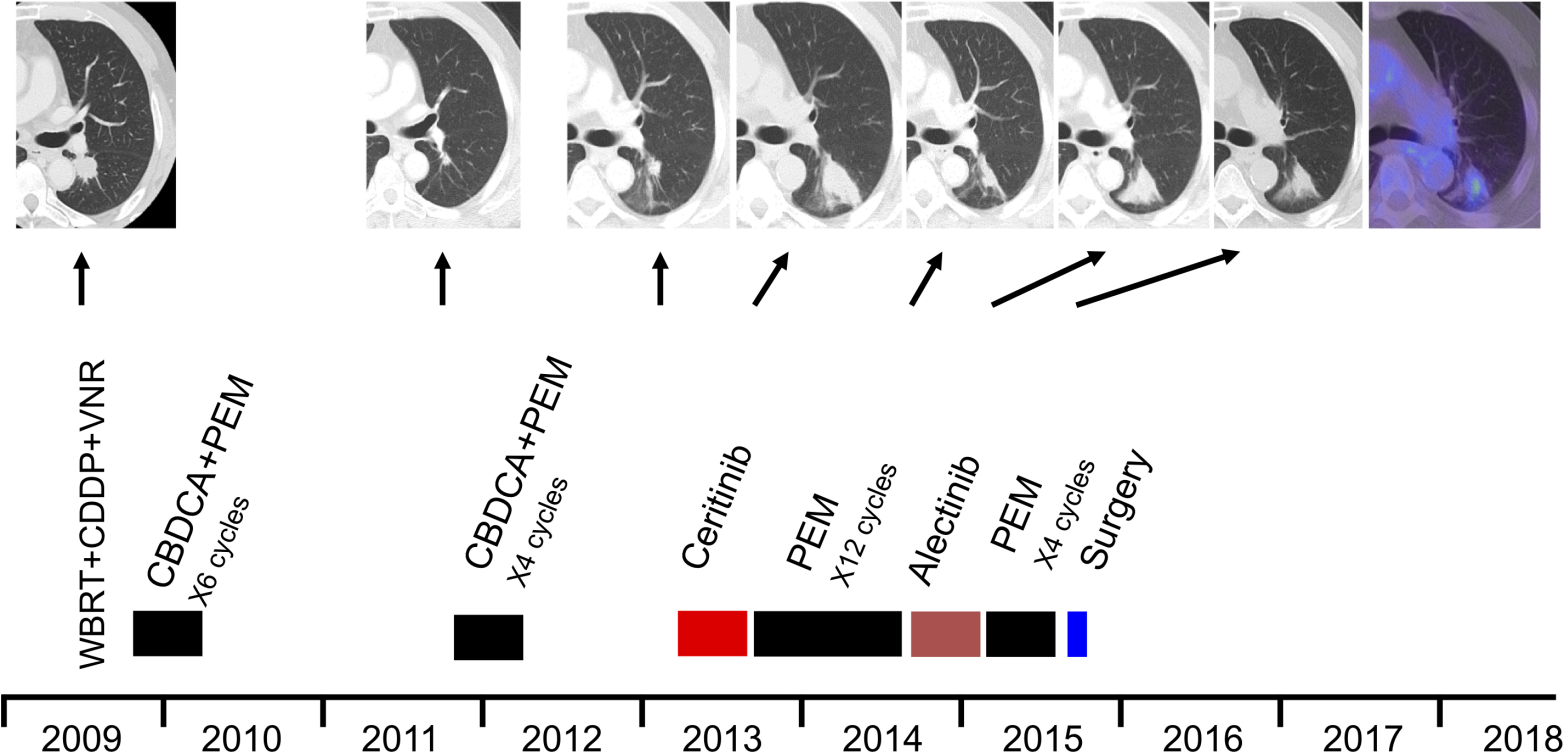
Timeline of the clinical course and computed tomography images. The patient received whole‐brain radiotherapy, chemotherapy, and ALK‐tyrosine kinase inhibitor. After the fifth local progression, he received a left sublobar resection. He is still alive with treatment‐free remission for more than 3 years.

A 71‐year‐old never‐smoker man was diagnosed by cervical lymph‐node dissection with advanced lung adenocarcinoma with more than five metastases, including brain, bone, and lymph nodes (cT1cN3M1c based on the 8th edition of the TNM staging system) at 62 years of age (Fig. 1). His performance status (PS) was 0. He received whole‐brain radiotherapy (WBRT) and one cycle of chemotherapy consisting of cisplatin and vinorelbine (Fig. 2). He was referred to our hospital and received six cycles of carboplatin and pemetrexed with good partial response sustained for 1.5 years. He developed first local progression of primary lung cancer of the left lower lobe and again received four cycles of carboplatin and pemetrexed with stable disease for more than 1 year. After developing a second local regrowth, he was enrolled into the industry‐initiated phase II clinical trial (CDLK378A2203) with ceritinib as a cervical lymph node specimen was positive for ALK rearrangement by fluorescence in situ hybridization (FISH). Eight months later, he developed a third local progression and then received 12 cycles of pemetrexed with partial response for 10 months, followed by treatment with alectinib because of its approval in Japan. Five months later, he developed a fourth local regrowth and received an additional six cycles of pemetrexed with minor response for 5 months. As magnetic resonance imaging (MRI) of the brain showed no abnormality, positron emission tomography (PET)‐computed tomography (CT) only showed hypermetabolic activity of the enlarged primary lung cancer, and he demonstrated easy fatigability associated with long‐term treatments, he decided to undergo sublobar resection and resection of accessible left hilar and left main bronchus lymph nodes. The pathological stage was ypT1aN0M0, stage IA with therapy effect grade 2a. RNAs extracted from a resected tumour showed a 75‐bp insertion between exons 24 and 25 besides EML4‐ALK (Echinoderm microtubule‐associated protein‐like 4‐anaplastic lymphoma kinase) rearrangement. Despite no further treatment, he is still alive for more than 3 years without recurrence.

## Discussion

A case of successful salvage surgery following multimodal therapy in advanced ALK‐rearranged lung adenocarcinoma is described. The chemotherapeutic activity of pemetrexed and durable responses to ALK‐TKIs, such as crizotinib, ceritinib, alectinib, and lorlatinib, can produce an median survival time (MST) of around 5 years in advanced ALK‐positive NSCLC 3, almost equivalent to the survival rate after surgical treatment of stage II NSCLC patients. Although the clinical features of the present case are the long duration of response, complete remission of metastatic sites with chemotherapy, and repeated local regrowth of the primary lung cancer, the effective treatments against ALK‐ rearranged lung cancer might eradicate most of cancer cells but fails to destroy drug‐resistant cancer cells. Therefore, a 75‐bp insertion between exons 24 and 25 besides EML4‐ALK rearrangement of resected primary lung cancer after second‐generation ALK‐TKI treatment may reflect a possible resistant mutation. Surgical resection of recurrent or persistent tumour after effective systemic treatments seems important for survival and might produce TFR and/or a cure, especially in a highly selected subgroup of ALK‐positive lung cancer patients.

Clinical T1‐2N0‐1 NSCLC patients with a single‐organ metastatic lesion are good candidates for surgical resection in a radical approach to disseminated NSCLC 2, 4. On the other hand, patients with more than five metastases have been reported to be classified in the most unfavourable subgroup for surgical resection among cases of advanced NSCLC 5. Recently, the indications for salvage surgery for stage IVb NSCLC are extending as TKI sometimes not only produces dramatic responses but also produces clinical downstagings to operable disease stages in advanced lung cancer patients harbouring genetic alterations 2, 4. However, the role of salvage thoracic surgery for stage IVb NSCLC with genetic alterations is unclear with regard to cure because a few reports have shown that initial staging could be unchanged even after dramatic radiographic response to EGFR‐TKI, as well as ALK‐TKI 4.

This might be the first representative case of successful salvage surgery for locally recurrent ALK‐positive NSCLC after response to chemotherapy, ceritinib, and alectinib in a patient with multiple organ metastases because this patient has achieved a long‐term treatment‐free period (≥3 years) after the completion of salvage surgery and systemic treatments without evidence of recurrence. Therefore, we applied the concept of TFR in advanced lung cancer. The concept of TFR is a current treatment goal in chronic myeloid leukaemia (CML) with long‐lasting deep molecular response (DMR) to TKI 6. In general, the achievement of sustained DMR for ≥2 years after long‐term TKI treatment is the key point for TFR and one of the important criteria for TKI discontinuation. About half of the patients who stop TKI treatment remain treatment free, whereas the others usually recur molecularly within the first 6 months after TKI cessation, but they recover optimal responses on re‐treatment. In this regard, we would extend and/or modify the definitions of TFR in advanced lung cancer as we use the term TFR for a case with sustained complete response after surgical resection of a single recurrent or persistent tumour after effective systemic treatments for 6 years. Although there is no long‐term experience with TFR in advanced NSCLC, further cases need to be examined to justify TFR strategies.

In conclusion, multidisciplinary treatment planning is critical for stage IIIb–IV NSCLC patients in the era of highly effective treatments, including chemotherapy, molecular targeted therapy, and immunotherapy, and we need more information about the association between each genetic alteration and the significance of salvage surgery for a single recurrent or persistent tumour after effective systemic treatments.

### Disclosure Statement

Appropriate written informed consent was obtained for publication of this case report and accompanying images.
